# Implantable cardioverter–defibrillator in hypertrophic cardiomyopathy

**DOI:** 10.21542/gcsp.2018.31

**Published:** 2018-08-12

**Authors:** Diego Jimenez Sanchez, Ignacio Fernández Lozano

**Affiliations:** Hospital Universitario Puerta de Hierro-Majadahonda, Madrid, Spain

## Abstract

Sudden cardiac death (SCD) is the most devastating complication in hypertrophic cardiomyopathy (HCM). The implantable cardioverter–defibrillator (ICD) has proven to be effective in SCD prevention in several clinical scenarios. In HCM population, it has demonstrated to successfully abort life-threatening ventricular arrhythmias despite the extreme morphology characteristic of HCM, often with massive degrees of left ventricular hypertrophy and/or LV outflow tract obstruction. Studies showed a high rate of appropriate intervention in secondary prevention and in primary prevention of patients considered at high risk. This appropriate intervention rate is even more significant considering the young and otherwise healthy patients that compose HCM population. Since SCD incidence in HCM is relatively low, optimal identification of patients at high risk is crucial. Classical strategy of risk stratification based on clinical risk factors has several limitations and has proven to overestimate risk. A new risk prediction model that provides individual 5-year estimated risk appears to be superior to traditional models based on bivariate risk factors. Perioperative complications seem to be similar to those related to the implant of other cardiac devices, while long-term complications have been traditionally in the spotlight. HCM patients are considered more vulnerable to ICD-related complications and inappropriate ICD therapy because of their young age at implant and increased prevalence of atrial fibrillation, but long-term follow-up data on ICD-related complications in general practice is limited. The subcutaneous implantable cardioverter defibrillator seems to be a safe and effective alternative in HCM, although long-term data are scarce.

## Introduction

Hypertrophic cardiomyopathy is a genetically determined heart muscle disease, most often caused by mutations in one of the sarcomere proteins genes, that confers a very diverse natural history^1–3^. For the majority of patients with HCM, prognosis is good, compatible with normal longevity whereas for a subgroup of patients there is a risk for a number of adverse disease-related complications. Among them, sudden death is the most devastating, even more if we consider that it usually occurs in a young population and in the absence of previous symptoms as the first disease manifestation. In fact, HCM is the most common cause of SCD in young people and trained athletes^4–6^.

**Figure 1.**
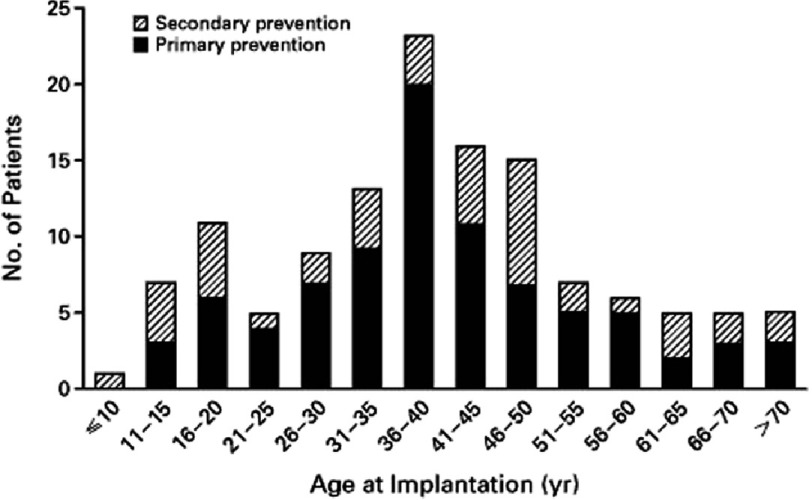
Age at the time of implantation of a defibrillator in 128 patients with hypertrophic cardiomyopathy who were judged to be at high risk for sudden death.

## Evidence of ICD in HCM

The ICD is widely accepted as a definitive treatment for the prevention of SCD. Initially it demonstrated prevention of SCD in ischemic heart disease patients^7–9^. Since no randomized trials of ICD therapy have been performed in patients with HCM, the indications for an ICD are derived from observational data that classifies patients in groups of risk based on their clinical features. With an incidence of SCD in HCM patients of 0.7% to 1.0% per year^10,11^, adequate risk stratification becomes a critical issue. The generally acknowledged non-invasive risk stratification strategy for primary prevention uses five clinical risk markers that have been defined in several retrospective and observational studies^12–15^. These markers are the presence of premature sudden death in relatives, history of unexplained syncope, the presence of non-sustained ventricular tachycardia (NSVT) in Holter ECG monitoring, the hypotensive response to exercise and massive hypertrophy (wall thickness ≥ 30 mm).

Another support for the efficacy of ICD therapy in HCM population is derived from the incidence of appropriate activation in patients who have had an ICD implanted. The study of Maron et al. ^16^, provided the first large evidence in this way of the utility of the ICD in high risk HCM population. In this retrospective, multicentre study, 128 HCM patients who had already implanted an ICD were enrolled, with a mean follow up period of 3.1 years. The average age of patients at the time of implantation was 40 years and more than 25 percent were under the age of 31 years (Figure 1). 34 percent of the defibrillators were implanted for secondary prevention, while the remaining 66 percent defibrillators were implanted prophylactically for primary prevention, in considered high-risk patients, consideration that was based on the generally accepted risk factors for SCD in HCM patients.

**Figure 2.**
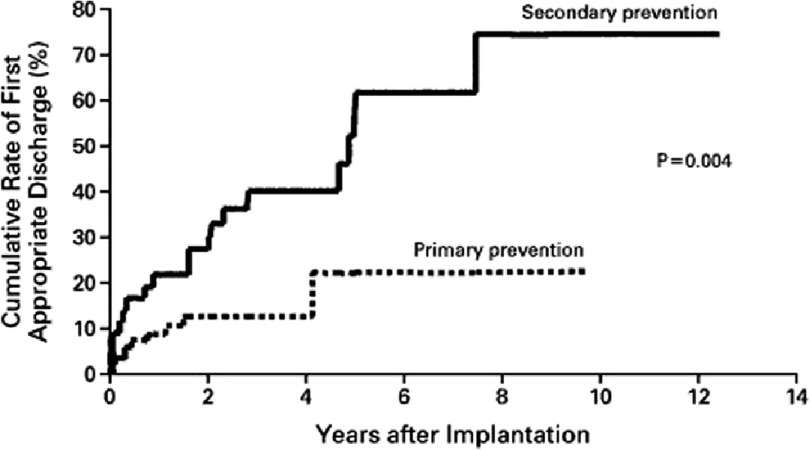
Estimated cumulative rates of first appropriate discharges, calculated separately for the 85 patients with defibrillators for primary prevention and the 43 patients with defibrillators for secondary prevention.

The rate of appropriate discharges for the secondary prevention group was 11 percent per year, while in the group of patients with devices implanted for primary prevention, the estimated rate of appropriate discharges was 5 percent per year. Cumulative rates of first appropriate discharges were significantly higher in the secondary-prevention group than in the primary-prevention group (*P* = 0.004). (Figure 2)

**Figure 3.**
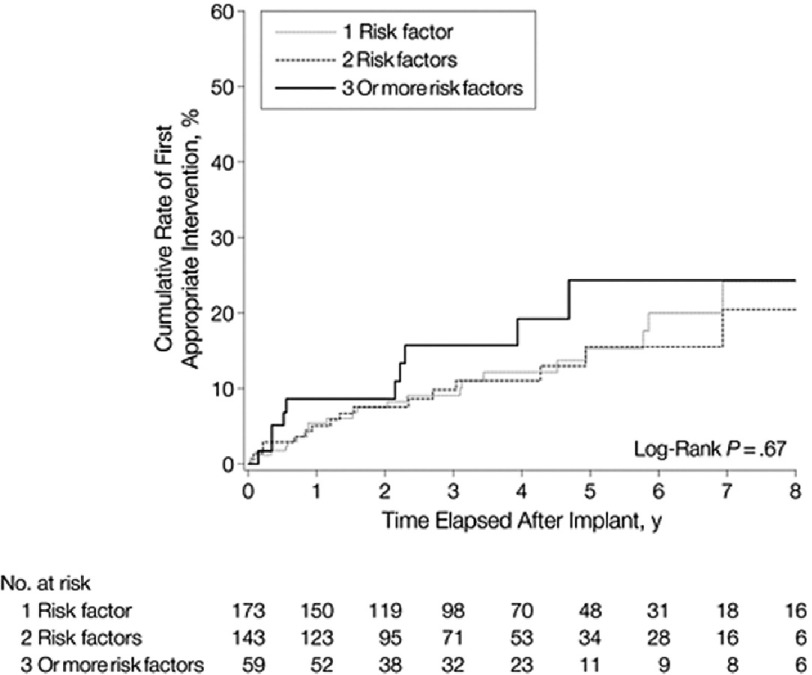
Cumulative rates for first appropriate implantable defibrillator intervention in patients with 1, 2, or 3 or more risk factors who had received devices for primary prevention.

A larger multicentre registry of 506 patients with HCM and an ICD (24 percent for secondary prevention) who were followed for an average of 3.7 years showed that 20 percent of patients received appropriate ICD interventions^17^. The rate of appropriate device activation was 10.6 percent per year when used for secondary prevention of SCD and 3.6 percent per year when used for primary prevention. Cumulative probability of discharge at 5 years was 39% in secondary prevention group (SD, 5%), being 17% (SD, 2%) in primary prevention group.

An important finding in this study was that 35% of the primary prevention patients who received appropriate device interventions for potentially lethal ventricular arrhythmias had been implanted with ICDs based on the presence of only one risk factor. In addition, no significant difference was observed with regard to the likelihood of appropriate ICD discharges among those HCM patients with 1, 2, or ≥3 high-risk markers (Figure 3).

Intervention rates, age at implant and percentage of primary prevention patients in both multicentre studies are consistent with those reported from other countries, including Spain^18,19^, Poland^20^, United Kingdom^21^, Australia^22^, and other U.S. centers^23^ (Table 1)

**Table 1:** Published experience with the ICD in patients with hypertrophic cardiomyopathy in the previous 10 years.

Author (Reference)	Year	Country	No. Patients	Mean Age at Implant	Appropriate Intervention no. (%)	Follow-Up (Years)	Intervention Rate (%)	Primary Prevention Patients (%)	Inappropriate Shocks (%)
Primo^20^	1998	Belgium; Spain	13	48	2 (15)	2.1	7.1	0	23
Maron^10^	2000	United States/ Europe	128	40	29 (23)	3.1	7.0	66	25
Begley^39^	2003	United States	132	34	27 (20)	4.8	4.2^*^	64	23
Jayatilleke^12^	2004	Australia	22	36	7 (32)	2.9	11.0	82	9
Przybylski^17^	2005	Poland	46	32	13 (28)	2.3	12.2	61	30
Marin^16^	2006	Spain	45	43	10 (22)	1.5	11.0	60	26
Kaski^21^	2007	United Kingdom	22	14	4 (18)	1.7	10.6	77	18
Maron^11^	2007	United States/ Australia/Europe	506	42	103 (20)	3.7	5.5	76	27

**Notes.**

* Criteria used for validating those arrhythmias triggering the implantable cardioverter-defibrillator (ICD) are not specified.

The presented data provides compelling support for the use of implantable defibrillators for secondary as well as for primary prevention in selected high-risk of patients. Nevertheless, the precise implications of the results showed, depend on the characteristics of the patients enrolled in the studies. This has particular relevance for the primary prevention strategy. Patients included in the studies came from selected HCM cohorts judged to be at high risk, thus the reported appropriate ICD discharge rates are not necessarily representative of what might be expected in a truly general HCM population with a more benign clinical profile.

**Figure 4.**
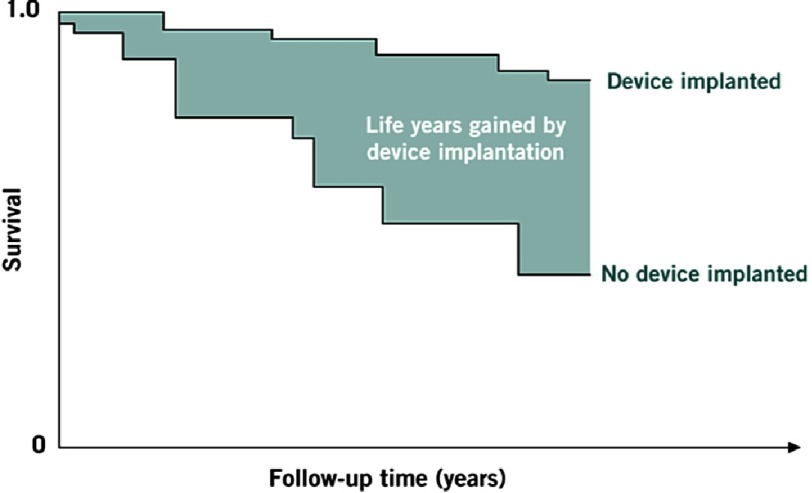
Life-years gained and survival curves. Number of lifeyears gained by device implantation is equal to area between Kaplan-Meier survival curves. As follow-up time increases, more area is revealed.

Another important fact supporting ICD therapy in HCM is the clinical and demographic profile of this population. In contrast with patients with an ICD indication for the consequences of coronary artery disease (CAD), HCM patients are much younger, often asymptomatic, they usually present with preserved ejection fraction and have much less comorbidities. For these reasons, HCM patients are exposed to a longer period of sudden death risk. Therefore, while annual appropriate intervention rates are lower in patients with HCM than in those with CAD, they are truly significant given the context of a much younger population usually free of limiting heart failure symptoms and other comorbidities. This long high risk period is especially important considering that the interval from ICD implant to first appropriate device intervention is variable, and often considerable in length, even as long as 10 years^16,17^. (Table 2) (Figure 4)

**Table 2:** ICD demographics in patients with HCM compared to coronary heart disease.

	CAD	HCM
Age at implant	∼65 years	∼40 years
Duration of risk	Short	Long
Myocardial substrate	Often compromised	Usually intact
Multi-organ disease	Frequent	Very rare
Ejection fraction as risk factor	Yes	No
Annual intervention rate	∼30%	∼5%

**Notes.**

CAD coronary artery disease ICD implantable cardioverter defibrillator HCM hypertrophic cardiomyopathy

### SCD risk stratification and ICD implant recommendations

Traditionally the recommendation for implanting an ICD has been done based on the presence of at least two major risk factors^24^. It is obvious that combination of risk factors in one patient make the decision more intuitive and easier for the clinician, but as presented before, initial data from high-risk HCM patients showed that appropriate device discharges occur with similar frequency in patients with 1, 2, or ≥ 3 non-invasive risk markers. However, a more recent study showed that risk increases with aggregation of risk factors and that the incidence of SCD in patients with a single risk factor is not significantly different from those without any^25^.

The approach of guiding ICD implant by risk stratification with clinical factors has led to algorithm-based recommendations^24,26,27^. This strategy has several limitations regarding the complex clinical scenarios involving gray-areas with respect to the presence, strength or number of risk factors. It does not account for the different effect size of individual risk factors and some risk factors such as LV wall thickness are treated as binary variables when they are associated with a continuous increase in risk.

Furthermore, classical risk factors can appear with a broad range of clinical presentations, which create difficulties to interpret them and uncertainty in the process of risk stratification. A characteristic example is the presence of unexplained syncope, which can mean very different things depending on the age of the patient, or clinical situation. It also occurs with NSVT findings in Holter monitoring, which may present as either an isolated brief run or multiple and/or prolonged bursts.

Validation of the proposed algorithms suggests that they overestimate risk, resulting in inappropriate prophylactic ICD implantation in a substantial number of patients^25^. Consequently, risk algorithms discriminate modestly between high and low-risk patients. As a result, other clinical features, such as myocardial fibrosis, LV apical aneurysms and the inheritance of multiple mutations, have been suggested as arbiters that can be used to guide ICD therapy in individuals who are at an intermediate risk, but there are few data to support this approach.

Another issue that has been a matter of concern and controversy is the risk of life-threatening arrhythmias or sudden death in HCM due to alcohol septal ablation (ASA). ASA, which represents an increasingly common and effective nonsurgical treatment for symptomatic obstructive HCM, consists in creating an iatrogenic myocardial infarction and therefore creating an iatrogenic scar. That could result in substrate for ventricular tachyarrhythmia and increase the incidence of sudden death.

In the larger ICD registry of patients with HCM and ICD^17^, appropriate discharge rates were 4-fold more common in patients with prior alcohol septal ablation (4 of 17 patients) compared with patients who underwent surgical myectomy (6 of 50 patients).

**Figure 5.**
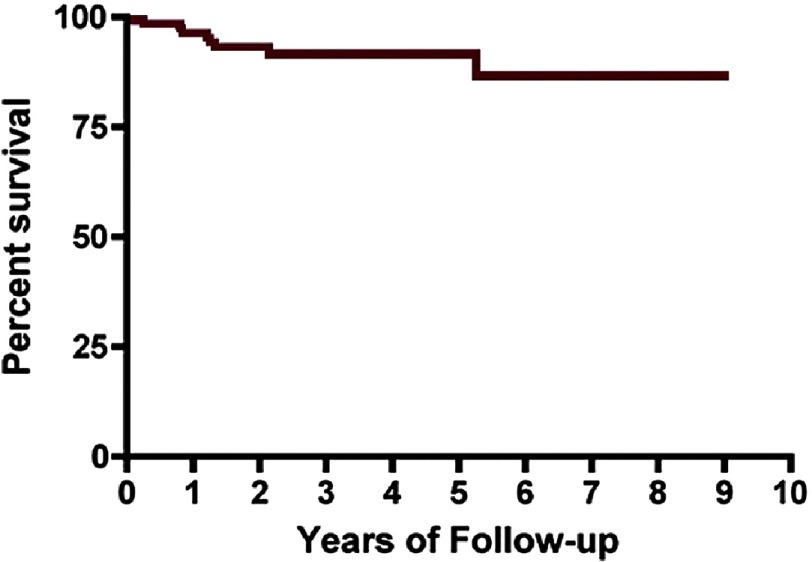
Kaplan–Meier curve for shock-free survival in patients after alcohol septal ablation.

With the purpose of assessing the effects of ASA on ventricular arrhythmias and SCD risk, a prospective study included 123 consecutive patients with obstructive HCM who underwent ASA and had an ICD implanted for primary prevention of SCD^28^. The estimated annual event rate was 2.8% over 3-year follow-up, which was low and even less than that reported previously for primary prevention of SCD in HCM. (Figure 5).

**Figure 6.**
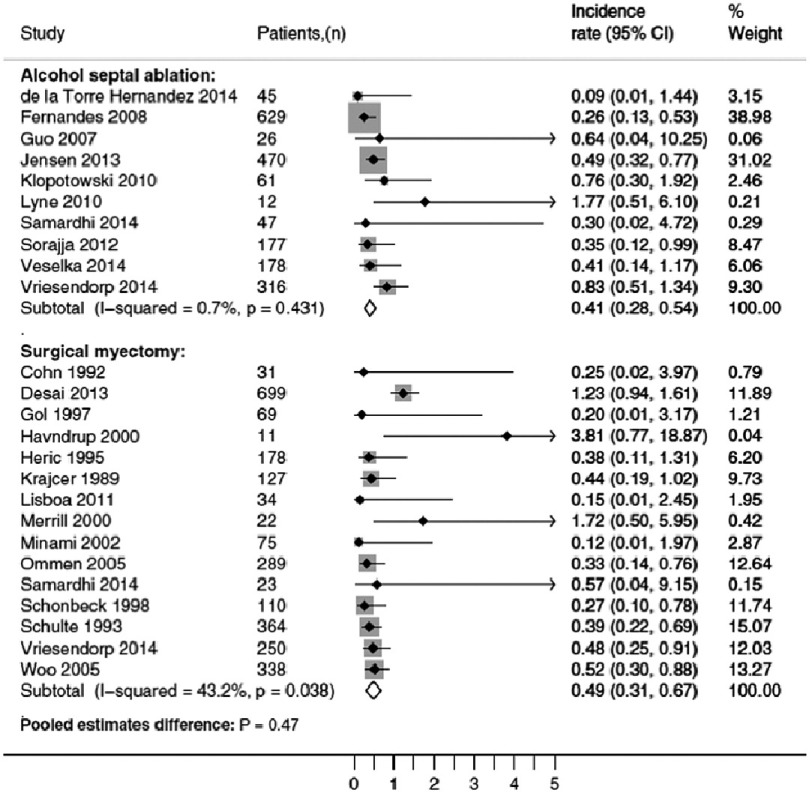
Forest plots and pooled estimates of (aborted) SCD rates after alcohol septal ablation and surgical myectomy, including ICD shocks.

A recent meta-analysis which pooled data from 11 ASA cohorts (2013 patients who underwent alcohol septal ablation) and 16 myectomy cohorts, showed no significant difference in long-term mortality, functional status, or aborted SCD between both groups^29^. (Figure 6)

With current evidence there is no supporting data showing that alcohol septal ablation increases the risk of sudden cardiac death.

With the aim of improving risk assessment and provide individualized risk estimation, a multicentre, retrospective, and longitudinal cohort study of 3,675 patients developed and validated a new SCD risk prediction model^30^. The HCM Risk-SCD model uses predictor variables that have been associated with an increased risk of sudden death in at least one published multivariable analysis, excluding abnormal blood pressure response as a risk marker. The model provides individualized 5-year risk. The broad patient inclusion criteria of the study mean that the model can be used in the majority of adult patients with HCM (paediatric patients under 16 were excluded), including those with mild disease identified during family screening.

The intention of the proposed SCD risk model is not to categorize patients into high or low risk groups with predefined therapeutic strategies, but to treat SCD risk as a continuum, to be interpreted within each patient’s clinical context.

**Figure 7.**
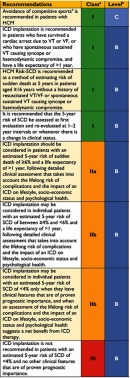
Recommendations on prevention of sudden cardiac death.

Validation studies have suggested that the new model is superior to traditional models based on bivariate risk factors^31,32^, and current clinical practice guidelines^33^, has included the HCM Risk-SCD model on their recommendations of prevention of sudden cardiac death as a method of estimating risk of SCD at 5 years in primary prevention (Figure 7).

### ICD in Spain

**Figure 8.**
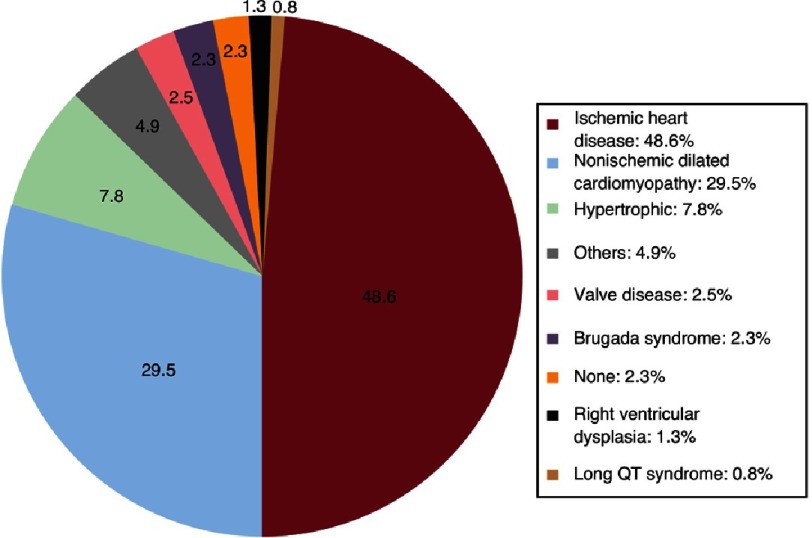
Types of heart disease prompting implantation (first implantations, sole diagnosis).

**Figure 9.**
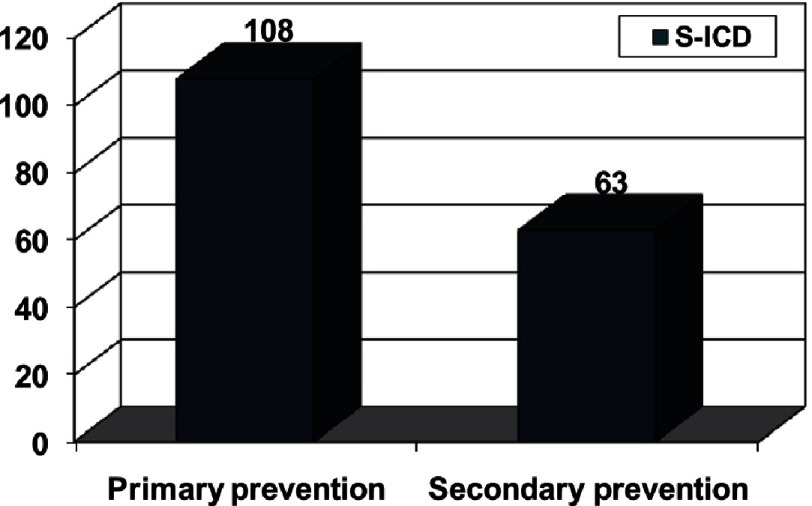


The Spanish 2016 Implantable Cardioverter-defibrillator Registry^34^ showed a reported implantation rate of 122 per million of population. HCM constituted the third leading cause of ICD implant in the registry after CAD and dilated cardiomyopathy. Subcutaneous defibrillator devices represented 6.4% of first implants (increasing from 2.4% in 2015) (Figures 8 and 9).

Spain was the country with the lowest number of implantations of all countries participating in the Eucomed (mean implantation rate of 320 per million population) in that year. The implantation rate per million population does not correspond with that expected from the clinical evidence, both in Spain and in other European countries.

Clearly, the country of residence plays a role in determining the proportion of HCM patients implanted with prophylactic ICDs. The variability among countries with respect to the number of ICD implants reflects with no doubt, differences in cultural perceptions, clinical decision making, economic matters and patient expectations that impact the threshold for recommending primary prevention devices to HCM patients.

### Paediatric population

Despite HCM predilection for young people, and an average age of patients included in the ICD studies around 40, with a substantial percentage of patients under the age of 30, data focusing on ICD therapy in paediatric patients with HCM are still few.

A large multicentre registry of children and adolescents with HCM and ICDs implanted for being considered at high risk was conducted, with the aim to assess criteria for implantation and clinical outcomes^35^. Decisions regarding ICD implantation were made at the discretion of managing clinicians, relying mainly on the risk stratification model based on risk factors established at that moment for the prevention of SCD in general patients with HCM.

**Figure 10.**
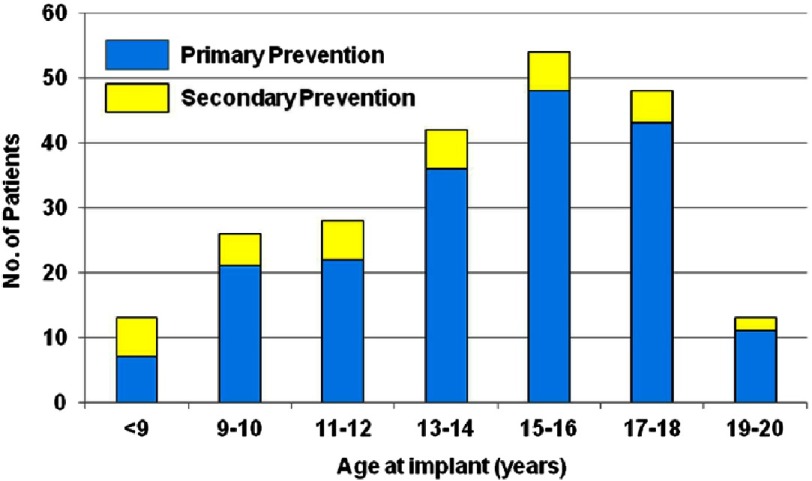
Patient age at defibrillator implantation. Shown for 224 pediatric patients with hypertrophic cardiomyopathy judged at high risk for sudden death who underwent implantation for primary or secondary prevention

**Figure 11.**
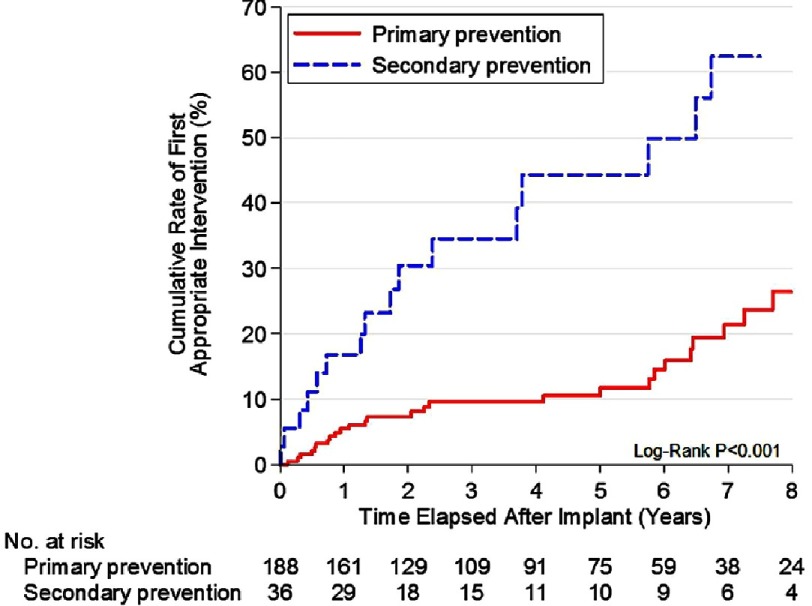
ICD intervention rates. Cumulative rates for first appropriate implantable cardioverter-defibrillator (ICD) intervention, shown separately for patients who underwent implantation for primary (*n* = 188) or secondary (*n* = 36) prevention. The rate of first appropriate ICD shock for secondary prevention exceeded that for primary prevention by 4-fold.

Rates reported of ICD intervention among the 224 children and adolescents included (84 percent placed for primary prevention) who were followed for an average of 4.3 years were similar to previously reported in adult registries. 43 patients (19 percent; 4.5 percent per year) received an appropriate ICD intervention. Appropriate interventions occurred in 14 percent of patients judged at increased risk for SCD by risk stratification, with cumulative 5-year probability of discharge of 12%. These rates predict the important impact of the therapy over the many years that these young and usually free from heart failure patients will be at risk. (Figures 10 and 11)

**Figure 12.**
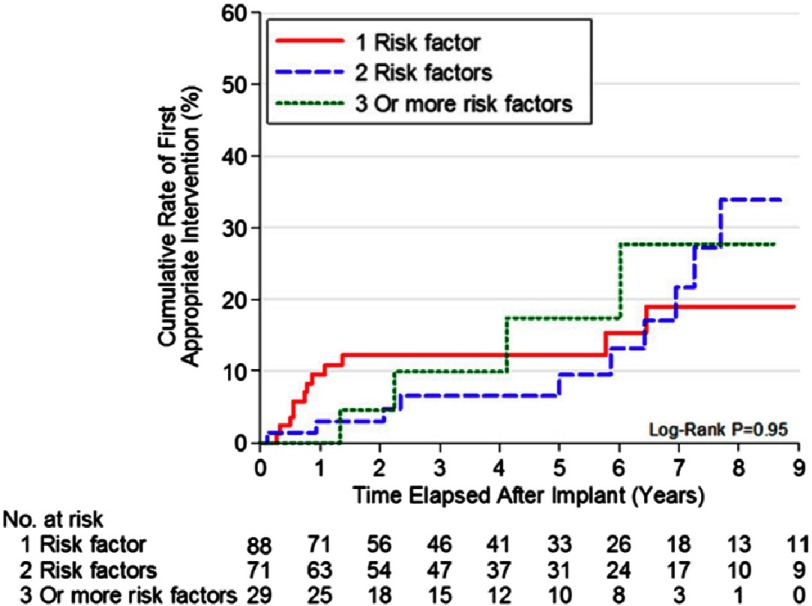
Number of sudden death risk factors and ICD interventions.

Similar to previous studies with adults, almost 50% of appropriate interventions were in patients who were considered high risk with the presence of only 1 risk factor. And again, there was no significant difference in the likelihood of appropriate ICD discharges among patients with 1, 2, or ≥ 3 conventional risk markers. Massive LVH was the risk factor most associated with appropriate interventions, and was found in about two-thirds of patients with discharges. That was consistent with findings in a study of 128 children <19 years old with HCM, in which septal thickness >190 percent above the 95th percentile for age and the sum of the R and S waves greater than 10 millivolts on ECG were found to be independent predictors of SCD^36^. (Table 3) (Figure 12)

**Table 3:** Clinical, echocardiographic, and demographic features in 224 children and adolescents with HCM who underwent ICD implantation for primary or secondary prevention.

		Primary Prevention		Secondary Prevention	
Characteristic	All Study Patients	Overall	≥1 Appropriate Intervention	Overall	≥1 Appropriate Intervention
Number of patients	224	188	26	36^§^	17
Age at implantation (yrs)	14.5 ± 3.6	14.7 ± 3.5^*^	14.0 ± 2.7^†^	12.9 ± 4.4	12.9 ± 4.5
Age at first ICD intervention (yrs)	16.4 ± 4.8	–	17.0 ± 3.4	–	15.6 ± 6.4
Male	151 (67%)	124 (66%)	16 (62%)^†^	27 (75%)	12 (71%)
Follow-Up duration (yrs)	4.3 ± 3.3	4.5 ± 3.3^*^	3.0 ± 2.7	3.5 ± 3.4	2.7 ± 2.7
NYHA class at implantation					
I	159 (71%)	126 (67%)	20 (77%)^†^	33 (92%)	16 (94%)
II	49 (22%)	46 (24%)^*^	3 (12%)^†^	3 (8%)	1 (6%)
III/IV	16 (7%)	16 (9%)^*^	3 (12%)^†^	0	0
NYHA class at last evaluation					
I	159 (71%)	133 (71%)	18 (69%)	26 (72%)	9 (53%)
II	47 (21%)	39 (21%)	6 (23%)	8 (19%)	6 (35%)
III/IV	18 (8%)	16 (9%)	2 (8%)	2 (6%)	2 (12%)
Maximal LV wall thickness (mm)	25.5 ± 9.8	26.0 ± 9.3	29.8 ± 11.4^‡^	22.7 ± 11.8	21.5 ± 9.4
LV end-diastolic dimension (mm)	40.2 ± 8.8	40.5 ± 8.6	41.8 ± 15.2^†^	38.7 ± 9.7	39.5 ± 10.5
Left atrial dimension (mm)	37.8 ± 9.2	37.9 ± 9.1	37.5±9.8^†^	37.6 ± 9.8	34.5 ± 9.8
LV outflow gradient at rest					
>30 mm Hg	54 (24%)	48 (26%)	10 (38%)^†^	6 (17%)	3 (18%)
<30 mm Hg	170 (76%)	140 (74%)	16 (62%)	30 (83%)	14 (82%)
Cardioactive medications at implantation	158 (71%)	135 (72%)	21 (81%)	23 (64%)	11 (65%)
Cardioactive medications at first appropriate intervention	36 (84%)	–	21 (81%)	–	15 (88%)
Ejection fraction <50%	9 (4%)	5 (3%)	1 (4%)	4 (11%)	3 (8%)

**Notes.**

Values are mean ± SD or *n* (%).

* Significant difference versus secondary prevention (*p* < 0.01).

† Variables unassociated with the likelihood of a primary prevention ICD appropriate intervention (*p* = 0.054 to 0.83).

‡ Significant difference versus secondary prevention appropriate interventions (*p* = 0.017).

§ Includes 10 patients with extreme LV hypertrophy with wall thickness ≥30 mm, of whom 4 had appropriate ICD shocks.

HCM hypertrophic cardiomyopathy ICD implantable cardioverter-defibrillator LV left ventricular NYHA New York Heart Association

The results presented show that the general principles of ICD use and efficacy in children are similar in many respects to those in adults. However, there are some unique considerations in paediatric patients, including the longevity of the device and lead, the size of the patient relative to the device, the increased physical activity, particularly in young children, and the different impact of ICD related complications. These issues need to be carefully considered when evaluating therapeutic options in children with HCM.

Current guidelines recommend that implantation of an ICD should be considered in children who have two or more major risk factors, not using HCM Risk-SCD model since it is only valid for patients older than 16, highlighting that in very young children, risk stratification is importantly limited by the lack of data^30,33^.

### Defibrillation thresholds

One of the vital aspects of ICD implantation is the demonstration that the myocardium can be reliably defibrillated. This is usually defined by the defibrillation threshold (DFT). The DFT is the lowest energy required to successfully defibrillate a patient, and a safety margin of>10 Joules (J) between the maximum output of the device and the DFT is usually recommended. Complications related to induction of ventricular fibrillation and DFT testing are uncommon.

Several studies tried to address the issue of identifying clinical predictors of increased defibrillation energy requirements with conflicting results^37–39^. HCM constitutes a disease with unique morphologic and hemodynamic features, such as extreme increase in left ventricular mass and dynamic obstruction to left ventricular outflow with elevated intraventricular pressures. As increased left ventricular mass has predicted elevated defibrillation threshold, patients with HCM are often considered to be at risk for increased defibrillation energy requirements^40^.

**Figure 13.**
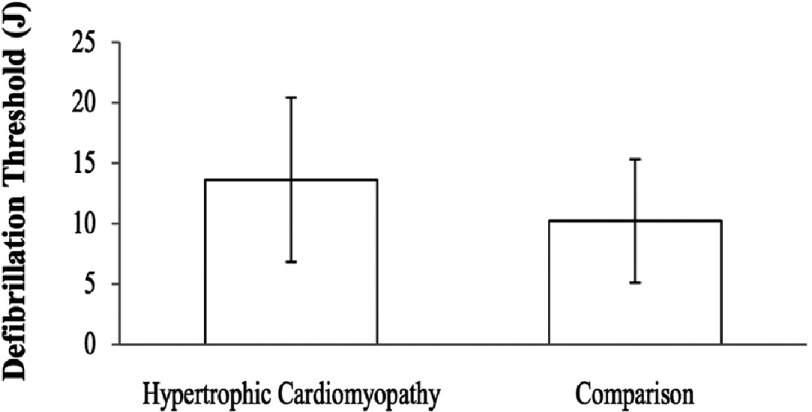
Average DFT was 13.6 ± 6.9 J in the HCM patients and 10.2 ± 5.5 J in the comparison group. The DFT difference was statistically significant ( *p* = 0.0037).

A retrospective review of patients who underwent ICD implantation and had DFT determined in one centre was performed to evaluate DFT testing in patients with HCM and clinical predictors of high DFT^41^. The study cohort consisted of 23 patients with HCM and the comparison group consisted of 294 patients within the same time period that had an ICD implanted for any indication other than HCM. The average DFT in the HCM group was higher (13.9 ± 7.0 J versus 9.8 ±5.1 J in the comparison group; *P* = 0.0004). In the HCM group, five of the 23 patients (22%) had a DFT ≥ 20 J compared to 19 of 294 comparison patients (6%). There was a significant correlation between DFT and left ventricle wall thickness in the HCM group (*r* = 0.44; *P* = 0.03) (Figure 13).

However, a prospective study of 89 consecutive patients with HCM and 600 historical control patients with ischemic or non-ischemic cardiomyopathy who underwent a standardized DFT protocol after ICD implant did not support the previous findings^42^. Mean DFT did not differ between groups. In the control group the mean DFT was 10.4 ± 5.8 J versus 11.2 ± 5.6 J in the HCM group ( *P* = 0.23). While the prevalence of high DFT (>15 J) tended to be greater in the HCM group (17% vs 12%), this did not reach statistical significance (*P* = 0.16). Subgroup analysis could not identify a parameter predicting HCM patients with high DFT.

**Figure 14.**
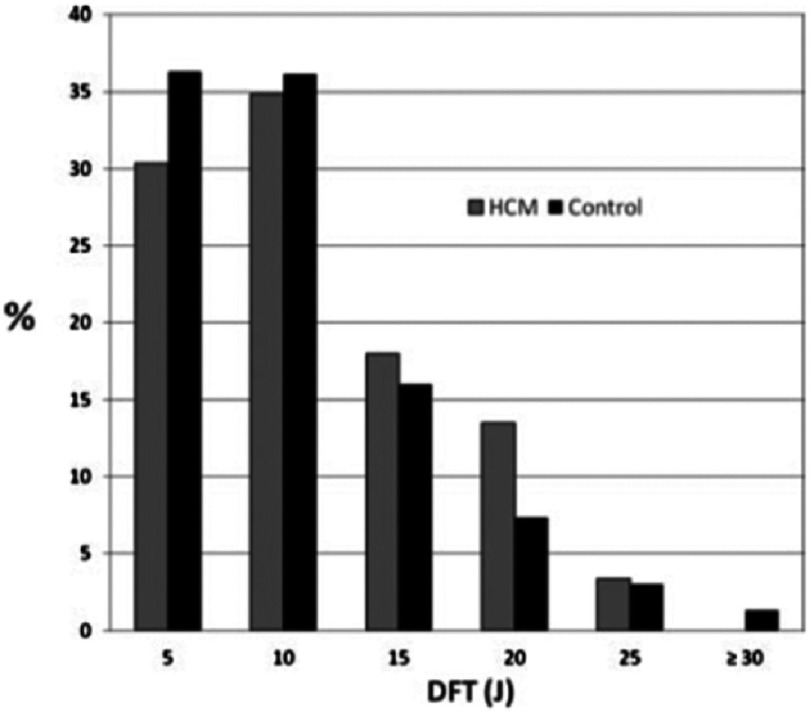
Comparison of defibrillation threshold (DFT) between control and hypertrophic cardiomyopathy (HCM) groups. Bars represent the percentage of patients in each group with DFT in the ranges shown.

These results suggested that the risk of unsuccessful defibrillation at implant in HCM is no different than the more typical ICD patients with left ventricular systolic dysfunction and that decision regarding the need to test defibrillation at implant should not be affected solely by the presence of HCM. (Figure 14) (Table 4)

**Table 4:** Patient characteristics for individuals with HCM and elevated (>15 J) and normal (≤ 15 J) DFT.

Characteristic	Elevated DFT	Normal DFT	*p* Value
Age (years ± s.d.)	51.5 ± 18.5	53.3 ± 14.8	0.69
Male sex, %	47	63	0.25
LVEF, % (±s.d.)	62.5 ± 7.17	64.8 ± 7.63	0.29
Septal thickness (cm)	2.45 ± 0.80	2.47 ± 0.62	0.92
LV mass, g (±s.d.)	364.4 ± 137.8	400.3 ± 143.8	0.42
LV mass index, g/cm^2^ (±s.d.)	188.2 ± 64.2	198.5 ± 60.0	0.59
QRS duration, ms (±s.d.)	136.9 ± 28.4	129.5 ± 28.1	0.37

**Notes.**

DFT defibrillation threshold HCM hypertrophic cardiomyopathy LV left ventricular LVEF left ventricular ejection fraction s.d. standard deviation

Current clinical practice guidelines emphasize that studies examining the role of defibrillation testing at the time of implantation are continuing, and recommend that until more data specific to HCM are available, defibrillation testing may be considered at the physician’s discretion^33^.

**Figure 15.**
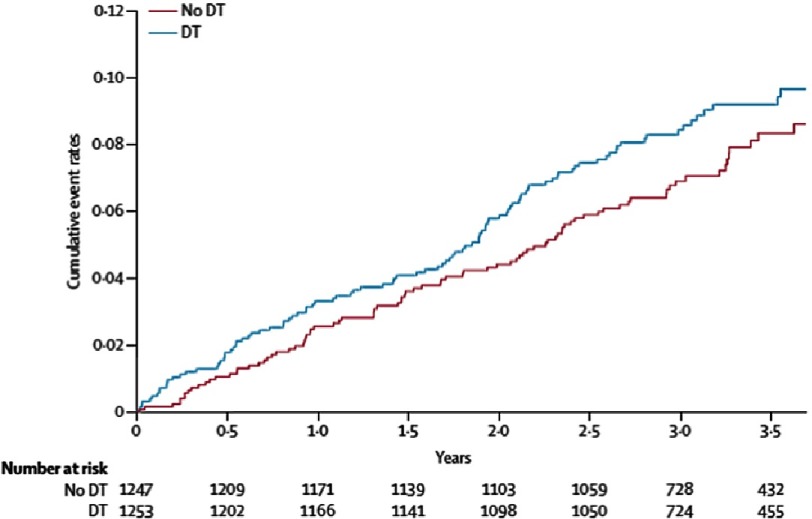
Failed appropriate shock or arrhythmic death. DT, debfibrillation testing. Mortality curve was constructed with the Kaplan–Meier method.

After guideline publication a larger, randomized, multicentre trial (SIMPLE trial), recruited 2,500 patients aged older than 18 years receiving their first ICD for standard indications at 85 hospitals in 18 countries worldwide^43^. Patients were randomly assigned (1:1) to have either defibrillation testing or not. HCM patients constituted 3.8% of the cohort. The primary outcome of arrhythmic death or failed appropriate shock occurred in fewer patients (90 [7% per year]) in the no-testing group than patients who did receive it (104 [8% per year]; HR 0.86, 95% CI [0.65–1.14]) (Figure 15)

A recently published substudy of SIMPLE trial including all HCM patients (*n* = 95) showed no difference in intraoperative defibrillation efficacy between patients with HCM and those with ICM/DCM^44^. Similarly, there was no significant difference between HCM patients with and those without defibrillation test in terms of clinical shock efficacy, or all-cause mortality.

These data throw light on the issue of DFT testing in HCM population, providing strong evidence that routine DFT testing does not seem to be associated with beneficial clinical effects in HCM patients.

### Device complications

The ICD good performance and success preventing SCD in HCM patients have been accompanied by clinical problems and device related complications. There are a wide variety of potential complications associated with ICD use, including those around the time of implantation as well as long-term over the life of the patient and the device.

Reported incidence of implant-related complications is similar to that found for permanent pacemakers and other implantable cardiac devices in the general population. Incidences of other early complications, including the need for acute lead revision or early device infection, are also low and similar to previously reported rates^45–47^.

Long-term complications mainly include system infection, lead malfunction or displacement and the delivery of inappropriate shocks. All these concerns are accentuated in a predominantly young patient cohort facing decades of future risk, who had probably been implanted with devices not specifically designed for them. Some authors suggested that patients with HCM would be more vulnerable to ICD-related complications and inappropriate ICD therapy because of young age at implant and increased prevalence of atrial fibrillation (AF)^45^. But long-term follow-up data on ICD-related complications in general practice is limited, hampering comparison of the inappropriate ICD intervention and ICD-related complication rates observed in different etiological groups.

A recent study compared defibrillation thresholds, perioperative complications, and long-term outcomes between patients with HCM (*n* = 95) and those with ischemic cardiomyopathy (ICM) or dilated cardiomyopathy (DCM) enrolled in the SIMPLE trial^44^. It did not find any difference in perioperative complications, and long-term outcomes between patients with HCM and those with ICM/DCM in the 3.1 ± 1.0 years of mean follow up duration of this study.

**Figure 16.**
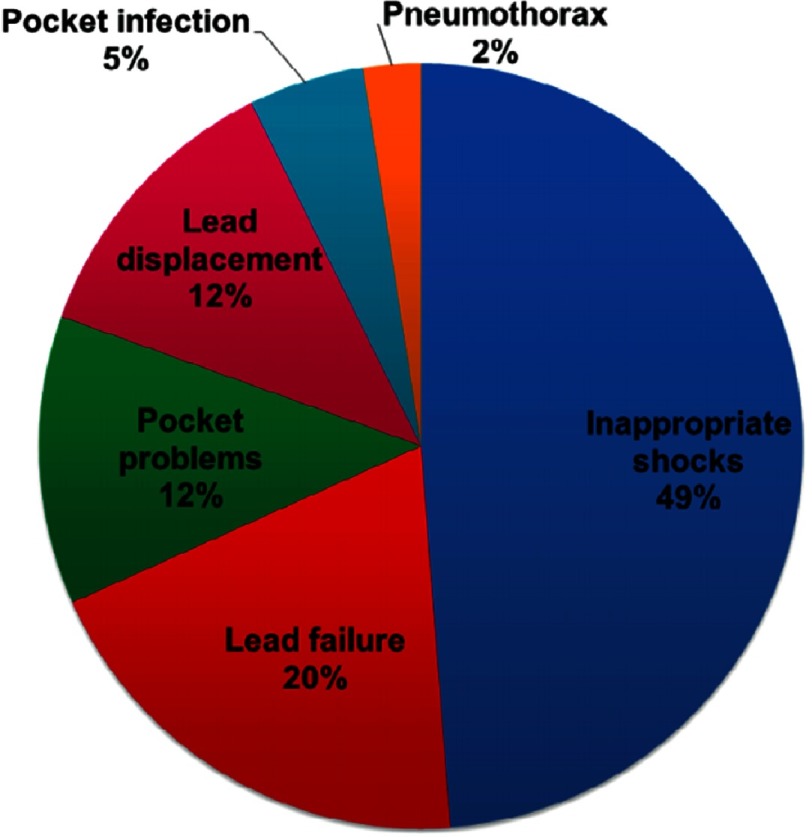
Distribution of adverse events in 134 HCM patients with an ICD.

A retrospective study of 134 patients was conducted to provide a complete overview of outcome and complications after ICD therapy in HCM patients^48^. Mean age was 47 with no patients under the age of 30 included. Adverse ICD events occurred in 36 patients (rate of 6.4%/year) including inappropriate therapies which comprised half of the events. (Figure 16)

Inappropriate ICD shocks occurred in 21 patients (3.7%/year) and were caused by AF in 50% of the cases. This study covered a 17-year period in which there was a considerable evolution in devices technology and expertise in implantation and programming, which could have affected the outcome. However, there was no difference in ICD-related complications or inappropriate interventions in ICDs implanted before or after 2007. Device settings, such as dual zone programming or longer cycle length for therapy zones, did not influence the frequency of inappropriate ICD shock. The amount of inappropriate ICD shocks was similar in patients with an atrial lead and in patients without it. (Table 5)

**Table 5:** Analysis of clinical variables associated with inappropriate ICD interventions (21 events in 134 patients with HCM).

	Univariable			Multivariable		
Predictor	HR	CI 95%	*P*	HR	CI 95%	*P*
Age at implantation, y	1.00	0.97–1.03	.9			
Male	0.8	0.3–1.9	.6			
NYHA III/IV	0.8	0.3–2.4	.7			
Atrial fibrillation	5.4	2.2–13.4	<.001	5.4	2.2-13.4	<.001
Coronary artery disease	2.5	0.3–19.8	.4			
Surgical myectomy	2.8	1.2–6.9	.02	3.1	1.2–7.6	.02
Alcohol septal ablation	0.4	0.09–1.6	.2			
Sudden death survivor	0.9	0.3–2.3	.8			
≥2 risk factors	0.6	0.2-1.5	.3			
Device-related:						
Single lead (VVI)	1.2	0.5–2.8	.7			
Atrial lead (DDD)	0.8	0.3–2.0	.7			
CRT-D	1.0	0.1–7.2	1.0			
Implantation before 2007	2.2	0.9–5.6	.09	2.4	0.9–6.0	.07
VF-zone only	1.2	0.5–2.9	.7			
VF-zone cycle length >290 ms	0.8	0.3–1.9	.6			

Similar rates for inappropriate shocks (4.8 and 4.9 %/year) and complications was reported in two different meta-analysis^49,50^ which included more recent studies.

Current data show that inappropriate shocks and other device related complications are not uncommon in this population. Furthermore, children and teenagers appear to have a higher incidence of lead fractures due to the strain placed on leads by their growth and development. Younger patients will also require multiple ICD generator changes throughout their life, which will increase the risk of device-related complications. This fact, combined with the prolonged risk time, the possibility of lost employment opportunities and limitations to quality of life, including the psychological burden of carrying an ICD, should not be underestimated in these patients.

Therefore, the decision to implant an ICD in a young patient with HCM should always be undertaken under careful discussion, and consideration of the potential complications and psychosocial issues.

### Subcutaneous implantable cardioverter defibrillator

The subcutaneous implantable cardioverter defibrillator (S-ICD), which is being now implanted in many countries worldwide, is comprised, as standard transvenous ICD (TV-ICD), of a pulse generator and a shocking lead. It has been developed in an attempt to minimize some of the complications of TV-ICD systems by avoiding endovascular access entirely.

The pulse generator is implanted in a subcutaneous pocket in a left lateral, mid-axillary thoracic position. The subcutaneous lead is tunneled from the pulse generator to a position along the parasternal margin.

As there are no formal guidelines for the selection of an S-ICD system, S-ICD are usually considered in young patients in order to avoid chronic leads and its complications, as well as in patients at high risk of systemic infections and in patients with challenging vascular access.

On the other hand, S-ICD devices provide neither antitachycardia pacing (ATP) nor continuous bradycardia pacing if necessary. For these reasons, an S-ICD should not be implanted in patients with the need for bradycardia pacing or for biventricular pacing in the setting of cardiac resynchronization therapy.

S-ICD devices as an alternative for TV-ICD have usually been considered in HCM population, since it is composed of young patients with a low theoretical risk for needing bradycardia pacing.

The risk of TV-ICD lead failure increases over time and is related to age and activity level. Lead failures occur more commonly in young active patients^51^, and it results in additional morbidity and mortality because of the need for additional transvenous leads, with or without lead extraction. This alternative therapy could prevent intravascular lead complications, which are even more significant hazards in these young patients, who potentially face at least 3–4 decades of device therapy. In fact, S-ICD implantation now has a class IIb recommendation in the European guidelines for HCM^33^; despite S-ICD efficacy and safety in this population are not clearly defined.

Several features of the disease were thought to potentially compromise efficacy and safety of S-ICD therapy; mainly left ventricular hypertrophy, which could increase DFT, and electrocardiogram abnormalities that could predispose to T-wave oversensing and inappropriate shocks.

Early data from small cohort studies supporting S-ICD use in patients with HCM are promising.

In a cohort of 27 patients with HCM considered for S-ICD implantation, 23 of 27 (85 percent) remained eligible following ECG screening implant, and the S-ICD terminated ventricular fibrillation (VF) with a 65J shock in all 15 implanted patients^52^. After a median follow-up of 17.5 (3–35) months, only 1 patient received an inappropriate shock attributable to oversensing, despite successful screening.

Outcomes of patients with HCM implanted with S-ICD were compared to non-HCM S-ICD recipients using pooled data from subjects enrolled in the EFFORTLESS Registry and US IDE study^53^.

HCM patients were younger, had preserved LVEF and were more likely to be implanted in primary prevention (Table 6). In the cohort of 872 patients (99 with HCM and with a median follow-up of 637 days), similar implantation success (98.9% of HCM and 98.5% of non-HCM patients) and one-year complication rates following S-ICD implantation were seen for patients with and without HCM. There were no significant differences between both groups in overall final shock conversion efficacy and inappropriate shocks.

**Figure 17.**
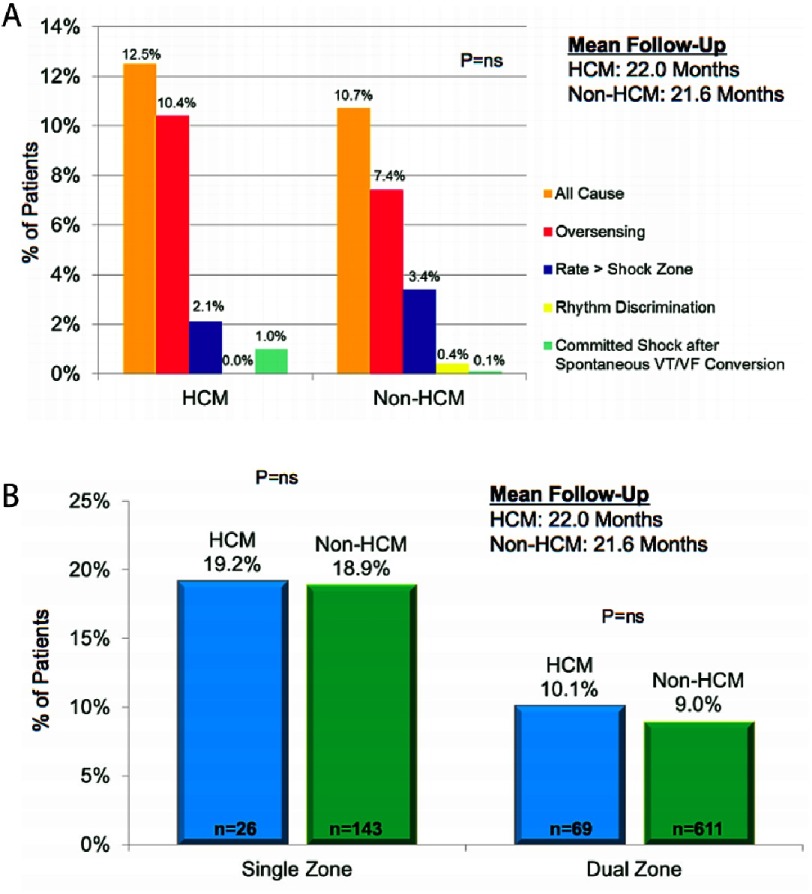
(A) Burden and aetiology of inappropriate shocks in the 2 groups. (Subcategories do not sum to the “All Cause” total because some patients had inappropriate shocks in more than one category). (B) Incidence of inappropriate shocks with dual and single zone programming (by patients).

In HCM, the majority of inappropriate shocks were caused by T wave oversensing with no significant difference with non-HCM patients. An equal proportion had supraventricular tachycardia (SVT), either sinus tachycardia or AF leading to an inappropriate therapy. As previously reported, dual-zone programming effectively reduced the inappropriate shock rate in both groups. (Figure 17)

**Table 6:** 

Category	HCM (*n* = 99)	Non-HCM (*n* = 773)	*P*
Age (y) (Range)	41.6 ± 15.8 (11–85.2)	51.3 ± 16.8 (7–88)	<.001
Male (%)	74.7	72.2	ns
Height (cm) (Range)	175.4 ± 9.3 (152–202)	174.5 ± 10.4 (137–208.0)	ns
Weight (kg) (Range)	86.8 ± 19.6 (34–153.3)	86 ± 23.2 (18–230.9)	ns
BMI (Range)	28.4 ± 6.2 (19–48.7)	28.2 ± 6.7 (15.2–69)	ns
LVEF (%) Range	65.1 ± 9.9 (34–86)	36.2 ± 15.6 (10–80)	<.001
Primary prevention (%)	87.9	67.5	<.001

An indirect comparison of S-ICD performance with TV-ICD in HCM patients was made, using data from a TV- ICD performance meta-analysis which included 2190 HCM patients^49^. An important difference noted was the lack of specific lead malfunctions in the S-ICD population versus malfunctions in 6.2 % of the meta-analysis patients.

Appropriate shock rates were about half in the S-ICD cohort compared with the TV-ICD cohort. Authors suggested shorter follow-up, a lower-risk population, or longer time to therapy with the S-ICD as possible explanations. Inappropriate shocks rates were similar between cohorts; however proportion of shocks that were inappropriate was high in the S-ICD cohort, given the low incidence of appropriate shocks. (Table 7)

**Table 7:** Comparison of S-ICD outcomes with an HCM TV-ICD meta-analysis.

Outcomes	Pooled HCM S-ICD (% pts)	Pooled non-HCM S-ICD (% pts)	HCM TV-ICD meta-analysis (% pts)	HCM S-ICD event rate (% pts/y)	HCM TV-ICD meta-analysis event rate (% pts/y)
Appropriate shocks	3	7.3	13.7 (9.9–17.5)	1.7	3.3 (2.2–4.4)
Inappropriate shocks	12.5	10.7	19 (12.6–25.4)	6.9	4.8 (2.2–6.7)
Complications					
Infection	2	1.6	3.1 (1.2-5)	1.1	0.6 (0.1-1)
Erosion	0	1.4			
Lead displacement	1	0.5	2.7 (1.6–3.9)	0.6	1.5 (0.9–1.1)
Lead displacement/malposition/ suboptimal system position	3	1.8	2.7 (1.6–3.9)	1.5	1.5 (0.9–1.1)
Lead malfunction	0	0	6.2 (4.1–8.3)	0	1 (0.5–1.4)
Conversion success rates					
Induced VT/VF	98.9	98.5			
Spontaneous VT/VF	100	98			

**Notes.**

HCM hypertrophic cardiomyopathy Pts patients S-ICD subcutaneous implantable cardioverter-defibrillator TV-ICD transvenous implantable cardioverter-defibrillator VF ventricular fibrillation VT ventricular tachycardia

**Figure 18.**
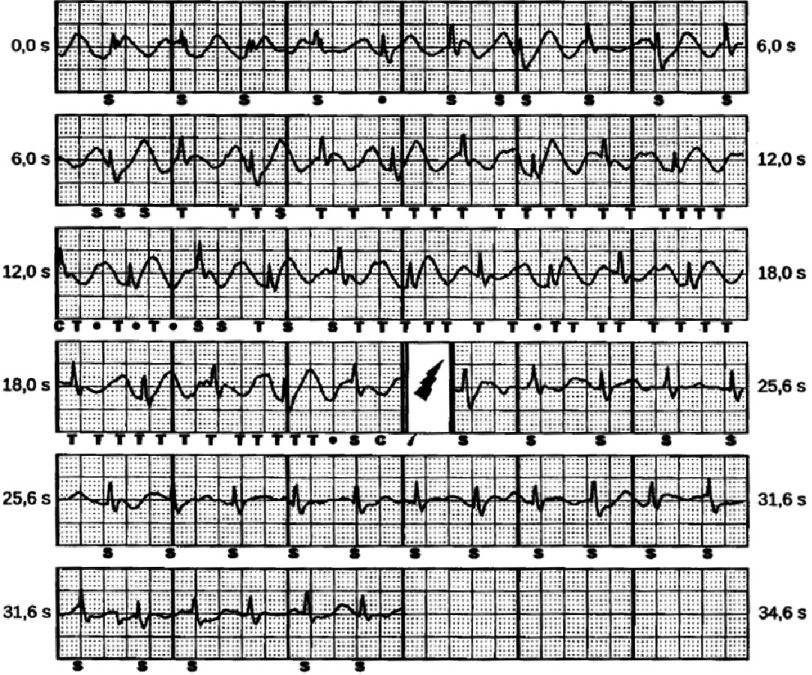


As we have seen, T-wave oversensing is the main cause for inappropriate shocks and recent improvements in the S-ICD discrimination algorithm have been shown to avoid inappropriate charging due to this problem. In contrast, TV-ICD systems are challenged by SVTs with rates in the therapy zone resulting in defibrillation. (Figure 18)

Authors suggested that the low incidence of inappropriate shocks for SVTs may reflect better specificity of the S-ICD discrimination algorithm and the use of dual-zone programming, added to the more prolonged detection and therapy times in the S-ICD versus standard TV-ICDs.

But most data from TV-ICD therapy used in this comparison is extracted from studies performed prior to publication of MADIT-RIT trial^54^, and modern programming may not be reflected.

## Conclusions

The ICD has proved to be a safe and effective therapy preventing SCD in HCM patients, which constitutes probably the major disease related concern. It reliably aborts life-threatening ventricular arrhythmias in those HCM patients judged to be at high risk for SCD, without needing to perform DFT testing routinely. There is no doubt of ICD indication in patients who were resuscitated from cardiac arrest or who had prior ventricular arrhythmias. For primary prevention, although there is no perfect risk stratification strategy, HCM Risk-SCD model seems to be the best available tool for identifying adult patients at risk for SCD. For paediatric patients, ICD implant should be considered with the concurrence of two major risk factors, considering that the potential life-saving performance of ICD devices must be weighted individually against concerns regarding device complications and psychosocial issues. ICD complications are not uncommon and they have more important implications in the particular setting of HCM patients. S-ICD seems to be an effective alternative for traditional TV-ICD, but long-term data of efficacy and safety are still needed. It is currently unclear how the lack of ATP will impact on HCM patient S-ICD shock rates and how important the problem of inappropriate shocks may become, considering the programming limitations of these devices.
